# Evolution of the Florida Pediatric Bone Marrow Transplant and Cell Therapy Consortium (FPBCC): A Statewide Initiative Toward Improving Transplant Outcomes

**DOI:** 10.1111/petr.70059

**Published:** 2025-03-06

**Authors:** Warren Alperstein, Jin‐Ju Lee, Deepakbabu Chellapandian, Natalie Booth, Jorge Galvez‐Silva, Michael Joyce, Jordan Milner, Paul Castillo, Reema Kashif, Mansi Dalal, John Ligon, David Crawford, Minelys M. Alicea Marrero, Jessica Peters, Biljana Horn, Edward Dela Ziga

**Affiliations:** ^1^ Pediatric Hematology and Oncology University of Miami School of Medicine Miami Florida USA; ^2^ Pediatric BMT and Cell Therapy University of Florida Gainesville Florida USA; ^3^ Blood and Marrow Transplant Program, Cancer and Blood Disorders Institute Johns Hopkins all Children's Hospital St. Petersburg Florida USA; ^4^ Nicklaus Children's Hospital Miami Florida USA; ^5^ Nemours Children's Health and Wolfson Children's Hospital Jacksonville Florida USA

**Keywords:** bone marrow transplantation and cell therapy consortium, effectiveness, pediatric, survival

## Abstract

**Background:**

Florida Pediatric Bone Marrow Transplant and Cell Therapy Consortium (FPBCC) was formed in 2018 by five pediatric transplant programs in Florida. The key objectives of the consortium are to improve outcomes for children undergoing HSCT through collaboration among centers, data sharing, implementation of best practices, QI projects, and prospective clinical trials. The first step in that process was to analyze HSCT outcomes from all participating centers and identify areas for improvement. In this report, we describe the effectiveness of the activities of this consortium, focused on improving patients' outcomes.

**Methods:**

A retrospective data review of allogeneic transplant 1‐year survival, obtained from the annual CIBMTR report, from the five FPBCC centers was compared to survival from 38 other pediatric centers in the country over two periods: preconsortium establishment, from 2016 to 2018, and postconsortium establishment, from 2019 to 2021. Of the 38 other pediatric centers, 22 were defined as small, similar to consortium centers by number of transplants (20–70 first allogeneic transplants per center in a 3‐year period) and 16 were larger centers (> 71 first allogeneic transplants per center in a 3‐year period).

**Results:**

The 1‐year posttransplant survival for the FPBCC centers significantly improved from 77.5% (2016–2018) to 89.5% (2019–2021; *p* = 0.0313). During the same respective time periods, other small centers improved from 82.4% to 87.9% (*p* = 0.0059), and large centers maintained stable survival at 85.6%–85.4% (*p* = 0.2676).

**Conclusions:**

There was a substantial improvement in the 1‐year survival of allogeneic transplant recipients treated in FPBCC centers, achieved after the initiation of consortium activities. Within a 3‐year period, consortium centers, which had a lower starting point, reached 1‐year survival comparable to that of other small and large centers. A significant improvement in survival, although a lesser percentage of change, was seen in other programs of similar size across the country, but not in larger programs. We consider that the magnitude of improvement in survival (12% points or 4% per year), which was not seen among other programs, attests to the effectiveness of consortium activities. A blueprint for improvement in outcomes established by the FPBCC can be shared with other programs around the world that strive to improve posttransplant survival.

## Graphical abstract

Survival after allogeneic hematopoietic stem cell transplants (aHSCTs) in the state of Florida since the creation of a state pediatric HSCT consortium has improved. This is significant because it demonstrates that small‐sized pediatric HSCT centers can work together in a consortium to improve outcomes similar to larger centers.

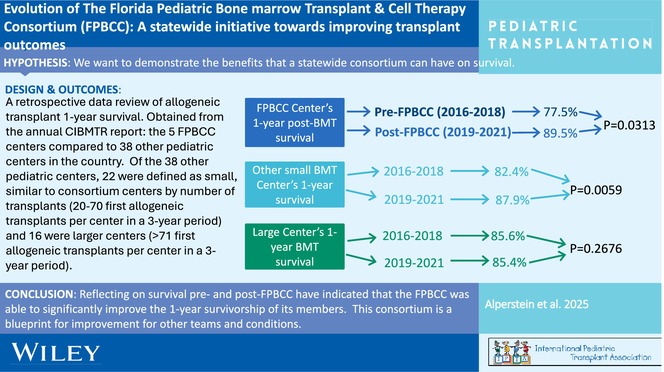

## 1 Introduction

Bone marrow transplantation (BMT) and cell therapies (CT) are complex, life‐saving procedures for many pediatric hematologic, oncologic, metabolic, and immunologic disorders. Children undergoing BMT and/or CT require care in specialized centers. The costs and the burden of care for these procedures are significant. At the time of initiation of the Florida Pediatric Bone Marrow Transplant (BMT) and Cell Therapy (CT) Consortium (FPBCC), there were six BMT/CT centers in the state of Florida, providing services to approximately 4.1 million children under the age of 18 years. The six centers combined performed approximately 80 allogeneic and 50 autologous transplants annually (BMT Infonet data 2014–2015). The 1‐year survival of children undergoing BMT in Florida was in line with expected survival rates; however, it was lower than in the nation's leading pediatric BMT centers. Having small programs distributed across the state improves access to care and it may improve the patient's experience; however, it is described that survival is better when transplants are done in centers with disease‐specific expertise, which tend to perform a larger number of transplants annually [1, 2, 3, 4]. In addition to the lack of disease‐specific expertise, due to the small number of transplants at each institution, analyses of individual centers' outcomes are skewed due to the small number of patients and are often insufficient for data‐driven implementation of practice change.

To overcome some of the disadvantages of small BMT/CT centers, we have organized a state‐wide pediatric consortium focused on the improvement of pediatric BMT/CT outcomes in Florida through clinical collaboration, data sharing, implementation of best practices, QI projects, and prospective clinical trials.

To evaluate the effectiveness of consortium activities, we analyzed and reported the change in CIBMTR‐reported 1‐year survival outcomes of centers participating in the consortium for the period immediately prior to consortium activities (2016–2018) and for the subsequent 3‐year period (2019–2021), in order to compare the change in FPBCC outcomes to other pediatric transplant programs in the US that were not participating in the outcomes‐oriented consortium.

## 2 Methods

### 2.1 Description of FPBCC Activities Targeting Improvement of Outcomes

Activities related to organizing FPBCC were initiated in April 2018. Two phone conferences with representatives from five of six centers were held to establish the interest of BMT/CT programs in collaboration and foundation of the consortium, and to prepare for an in‐person meeting. On September 29, 2018, the first FPBCC in‐person meeting, with representatives from the five participating centers, was held in St. Petersburg, FL. At that meeting, consortium goals were defined as follows: (1) provide administrative and bioinformatics infrastructure for data sharing and statistical analyses, (2) facilitate collaboration among members by organizing regular video conferences and annual meetings, (3) support identification of BMT/CT‐specific quality indicators and support QI projects leading to the implementation of best clinical practices, and (4) facilitate the development of investigator‐initiated clinical trials (IIT) in the field of BMT/CT. A Florida Department of Health Bankhead‐Coley (BHC) Cancer Research Program 2018–2019 grant application was submitted in the fall of 2018. Although the BHC grant was not awarded, the blueprint outlining proposed consortium activities in the grant application was followed. The consortium activities were funded through three Children's Miracle Network grants.

Five out of six pediatric BMT/CT programs in Florida signed data use agreements and memorandum of understanding describing the rules of participation in the consortium and data sharing and formed the FPBCC. All institutions obtained IRB approvals/exemptions for data sharing and for each of the retrospective analyses of pooled data that were performed by the consortium.

Activities of the consortium included: (1) monthly 1‐h video conferences, which started in November 2018 and are ongoing. The meetings are open to physicians and all other interested BMT/CT staff. During these conferences, results of retrospective analyses, quality improvement projects, and preparations for a prospective study were discussed. The participants also discussed clinically challenging patients and shared their transplant‐related practices; (2) retrospective analyses were performed on pooled data. Each center downloaded its data from the CIBMTR platform and submitted it to the Consortium for joint analyses of outcomes. The goal of the retrospective analysis was to identify risk factors for survival and to identify areas for improvement. First, outcomes for a 3‐year cohort (2014–2016) were analyzed; subsequently, retrospective analyses were performed by disease category, including 10‐year data (2010–2019). The third retrospective study gathered additional data from centers related to outcomes of patients with severe aplastic anemia and data on disease‐free‐graft‐versus‐host free survival of patients transplanted for malignant disorders for a 5‐year period (2016–2020). Two quality improvement projects were implemented consortium wide. The first one was related to improvement in the accuracy of reporting of causes of death to the CIBMTR, and the second one introduced a change in donor selection criteria based on the results of survival by donor type from the 10‐year retrospective data; and (3) a prospective phase II clinical trial targeting high‐risk patients with hematologic malignancies was opened through the consortium and is currently enrolling patients.

**FIGURE 1:**
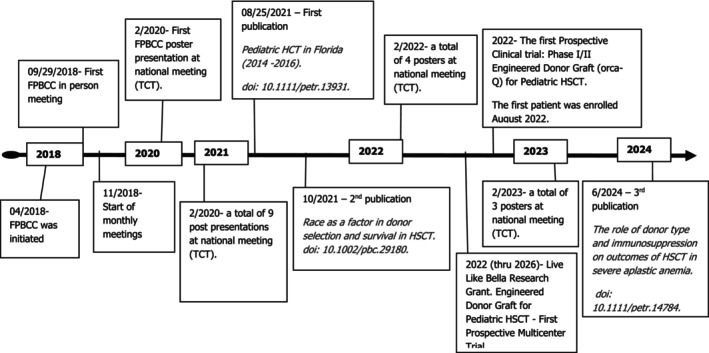
Timeline of consortium activities.

As prespecified in the consortium blueprint, the effectiveness of combined consortium activities was assessed by comparing 1‐year survival of patients receiving allogeneic transplant for a 3‐year period immediately prior to the initiation of consortium (2016–2018) and the 3‐year period after initiation of consortium activities (2019–2021). Figure 1 depicts consortium activity from inception [5, 6, 7].

### 2.2 Methods for the Analysis of the Effectiveness of FPBCC Activities

Data for the analysis were obtained from the CIBMTR annual Transplant Center‐Specific Survival Reports published in 2020 (for 2016–2018 period) and in 2023 (for 2019–2021 period). One center (UF), which reports combined pediatric/adult outcomes, provided their actual 1‐year survival of recipients of first allogeneic transplants because their data could not be obtained from the Annual Transplant Center‐Specific Reports. The mean predicted survival reported from the CIBMTR for the four Florida transplant centers reporting pediatric data was used for the entire FPBCC, assuming that patient characteristics were similar across the five Florida pediatric programs.

Five FPBCC centers and 38 other pediatric centers were compared to each other over the years 2016–2018 (preconsortium activities or pre) and 2019–2021 (postconsortium activities or post). The 38 other centers included all pediatric centers with > 20 allogeneic transplants over the 3‐year period and had outcomes reported for both periods. Pediatric transplant centers that report outcomes to CIBMTR together with adult data could not be included in this analysis. Subsequently, the 38 transplant centers used in comparison were divided into 22 small‐size centers (20–70 first allogeneic transplants per center in a 3‐year period) and 16 large‐size centers (≥ 71 first allogeneic transplants in a 3‐year period). All FPBCC centers belonged to the small‐size group.

Paired t‐tests were used to compare pre‐ and post‐1‐year survival of patients undergoing first allogeneic transplants, as well as the number of transplants. In addition, the predicted survival established by the CIBMTR was compared to the center's actual survival, and the difference between the two was tested by using the Mann–Whitney test. Kruskal–Wallis test was used to compare survival differences in FPBCC, other small, and large centers.

Descriptive statistics were used to summarize survival, differences between actual and predicted survival, and the number of transplants.

**FIGURE 2:**
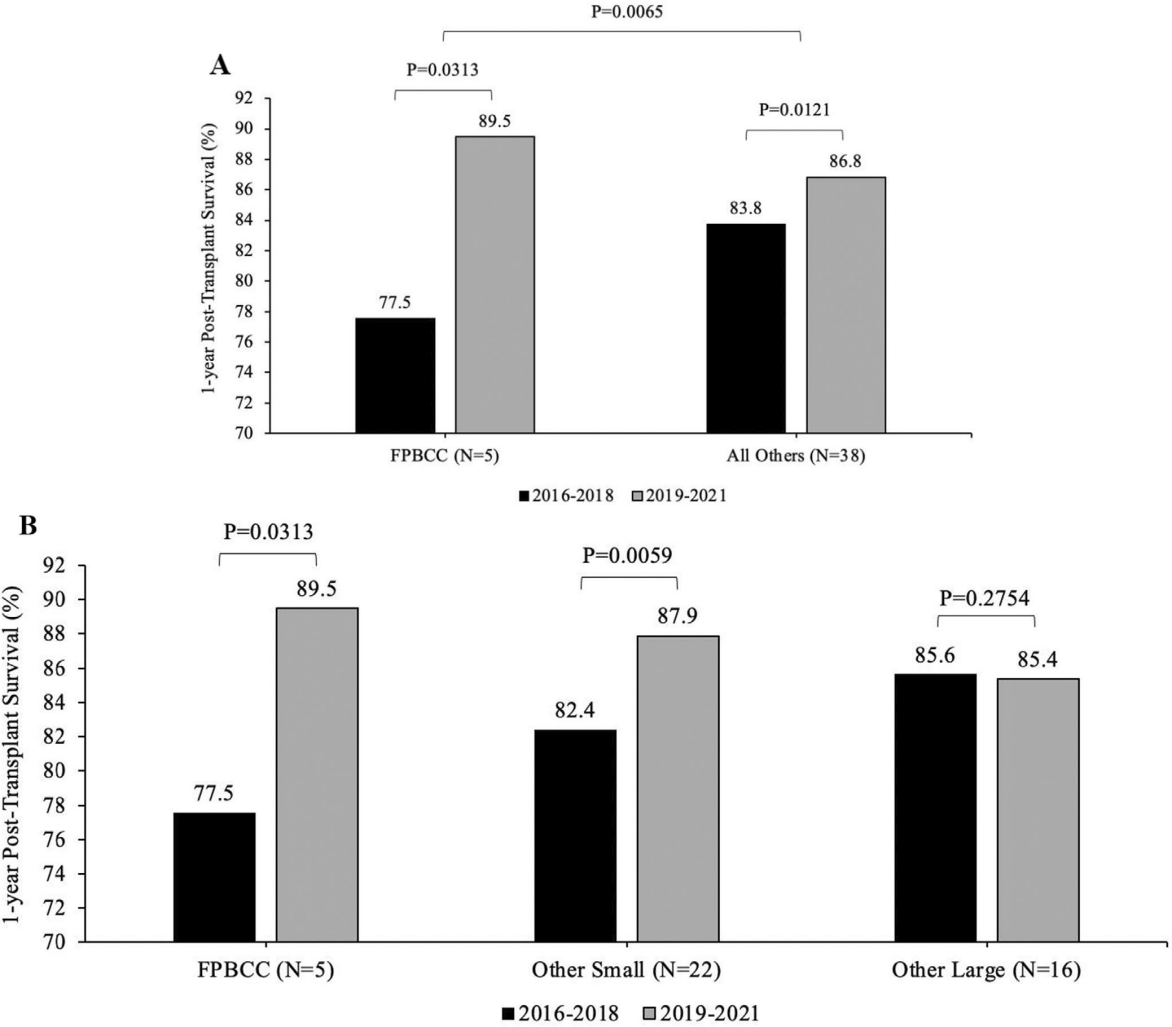
(A) One‐year posttransplant survival in 2016–2018 and 2019–2021. (B) One‐year posttransplant survival of patients treated in small and large centers.

**FIGURE 3:**
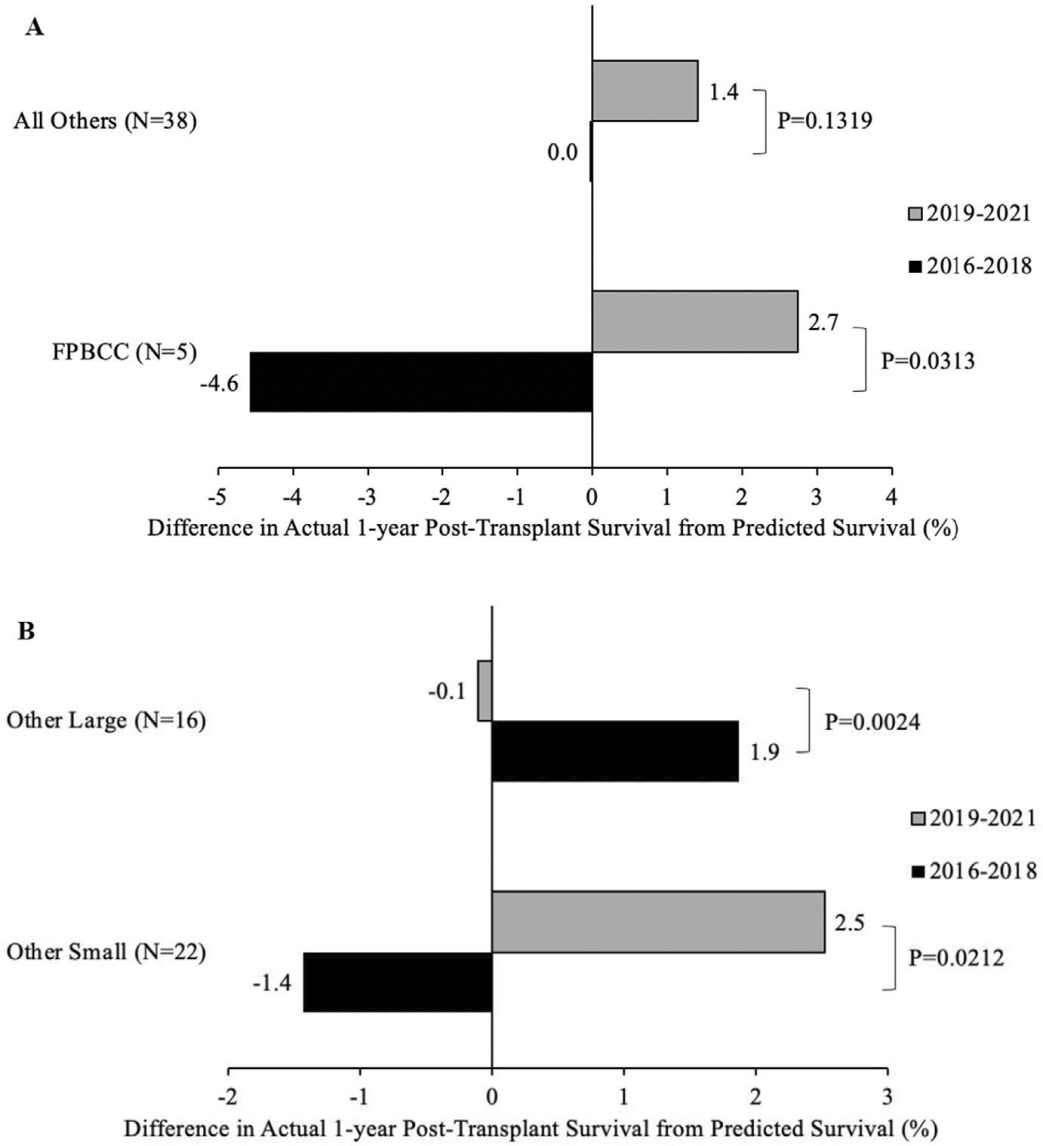
(A) Comparison of actual survival to predicted survival. (B) Comparison of actual survival to predicted survival for small and large centers.

**FIGURE 4:**
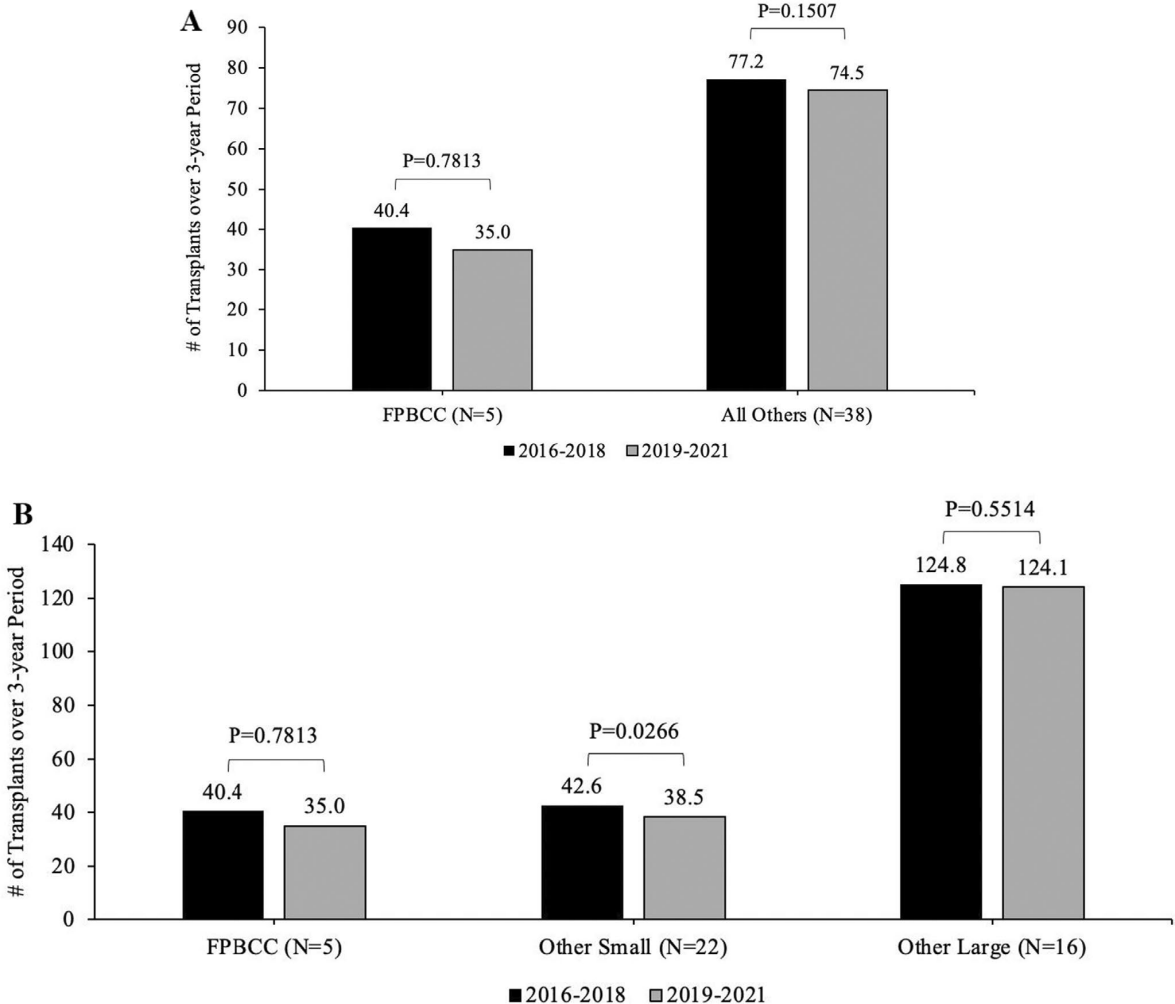
(A) Average number of transplants per center for 2016–2018 and 2019–2021 period. (B) Average number of transplants for small and large centers.

We present the following mean ± SE results:

1. Actual 1‐year survival during the two periods (pre and post) for FPBCC centers and all other centers (Figure 2A) and statistical testing for intragroup improvement and across the two groups improvement.
2. Actual 1‐year survival during the two periods (pre and post) FPBCC and other small and large centers with calculating *p*‐value for intracenter change as well as for change between FPBCC and small centers and FPBCC and large centers (Figure 2B).
3. Difference between the actual and predicted survival for FPBCC centers versus all other centers (Figure 3A) and FPBCC versus small centers, and large centers (Figure 3B) and statistical testing of the magnitude of that difference.
4. Mean ± SE of transplant numbers for the 3‐year period for FPBCC centers and all other centers (Figure 4A); and between FPBCC centers and small centers and large centers.

## 3 Results

### 3.1 Overall Survival in FPBCC and Other Programs

We compared the survival of five FPBCC centers with survival from the 38 other centers over the time periods before and after the initiation of FPBCC outcome improvement activities. Before the consortium activities, 1‐year posttransplant survival was 77.5% ± 4.8 for the five centers combined, and in the postperiod, the FPBCC survival was 89.5% ± 3.4. This improvement in survival over the 3‐year period was statistically significant, with a Wilcoxon signed rank test paired t‐test two‐tailed *p*‐value of 0.0313. The pre‐ and postsurvival for the other 38 pediatric centers was 83.8% ± 1.1 and 86.8% ± 0.8. That improvement over time was also statistically significant by paired t‐test, *p*‐value = 0.0121. When the improvement in survival among FPBCC centers was compared to the improvement in survival among the other 38 centers, the results were statistically significant with Mann–Whitney *p* = 0.0065, indicating a larger magnitude of improvement among FPBCC centers than among the other 38 pediatric centers (Figure 2).

The 3‐year overall survival pre‐ and postperiods for nonconsortium small centers were 82.4% ± 1.5, versus 87.9% ± 1.2, with the difference in improved survival being statistically significant *p* = 0.0059. The outcomes for large centers for the pre‐ and postperiod were 85.6% ± 1.3 and 85.4% ± 0.9, and this difference was not statistically significant with *p* = 0.2676. Survival differences among FPBCC, small, and large centers were significant with the Kruskal–Wallis *p*‐value = 0.0062.

Results are summarized in Figure 2A,B.

### 3.2 Difference Between Predicted Survival and Actual Survival in FPBCC and Other Centers

CIBMTR predicted survival and actual survival with intragroup and intergroup *p*‐value was presented for FPBCC centers and the other 38 centers (as well as the small and large centers). The FPBCC center that reported data together with the adult data lacked pediatric‐specific predicted survival data, necessitating an estimation based on the survival data from other FPBCC centers.

In the years 2016–2018, the five FPBCC centers had mean actual survival that was 4.6% ± 4.5 points lower than their predicted survival, while the nationwide data showed actual survival in alignment with their predicted survivals. In the 2019–2021 period, the actual survival was 2.7% ± 3.1 and 1.4% ± 0.8 higher than predicted in FPBCC centers and all other programs, respectively. The comparison of survival between the two time periods in FPBCC centers showed that survival was statistically significant at Wilcoxon signed rank *p* = 0.0313. All other centers did not have a significant change in actual and predicted survival between the time periods, as expected when all data are pooled as they define predicted survival.

During the years 2016–2018, other small centers had mean actual survival that was 1.4% ± 1.6 points lower than their predicted survival. In the subsequent period from 2019 to 2021, the actual survival rate among these smaller centers exceeded predictions by 2.5% ± 1.2. Meanwhile, during 2016–2018, larger centers outperformed their predicted survival rates by 1.9% ± 1.2. However, this performance saw a slight decline in the 2019–2021 period, with centers experiencing actual survival rates 0.1% ± 0.6 lower than their predicted survival. Notably, the comparison of survival rates between the two time periods for smaller centers yielded a statistically significant difference at Wilcoxon signed rank *p* = 0.0212. Similarly, larger centers also experienced a statistically significant decline in actual versus predicted survival between the time periods, with a Wilcoxon signed rank *p* = 0.0024.

### 3.3 Number of Transplants per 3‐Year Period

FPBCC centers had a 3‐year average of 40.4 ± 8.7 transplants per center over the first reporting period, while all other transplant centers had an average of 77.2 ± 8.4 transplants for the first period. In the second reporting period, FPBCC centers had an average of 35 ± 6.5 transplants, and other centers had 74.5 ± 8.8 transplants. The decline in the number of transplants in FPBCC centers and all other centers did not represent a statistically significant change.

When the change in the number of transplants was further evaluated by transplant size, small centers, excluding FPBCC centers, had a decline from 42.6 ± 3.8 to 38.5 ± 3.8 transplants over the two periods. This observed decline in transplant numbers seen in small centers was statistically significant (Wilcoxon signed rank *p* = 0.0266), while the large transplant centers did not have a significant decline in their numbers (124.8 ± 11.1 to 124.1 ± 12.1, Wilcoxon signed rank *p* = 0.4691).

## 4 Discussion

The FPBCC was established in 2018, as a response to the identification of lower HSCT survival rates in the different group member institutions in Florida. The 1‐year post‐HSCT survival rate (77.5%), although within an acceptable range, was lower than leading US pediatric centers. We recognized that the lower survival rate would allow for a faster improvement if appropriate interventions were implemented. The FPBCC proposed a set of activities, such as: monthly meetings, sharing of clinical experiences, data sharing, and identifying areas for improvement through retrospective analyses of outcomes. Although these are common sense activities, there were no previous measures of their ability to change survival outcomes. The significantly improved 1‐year post‐HSCT survival rate from 77.5% to 89.5% has been remarkable (*p* = 0.0313) and a surprise to participating FPBCC centers. The other small centers' 1‐year post‐HSCT survival improved from 82.4% to 87.9% (*p* = 0.0059), and other large centers had a stable 1‐year post‐HSCT survival rate at 85.6%–85.4%.

Interestingly, when looking at the actual 1‐year post‐HSCT survival rate compared to predicted survival based on CIBMTR data, the FPBCC centers were − 4.6% +/− 4.5 points lower than predicted in the pre‐FPBCC period. However, the FPBCC centers' survival improved compared to CIBMTR predicted survival data (+2.7% +/−3.1) during the second reporting period 2019–2021, which was statistically significant with a *p*‐value of 0.0313. When looking at the FPBCC data compared to other centers, the other smaller centers also had an improved actual survival compared to predicted (−1.4% to 2.5%, *p* = 0.0212), while other large centers did not have an improved survival rate compared to predicted (1.9% to −0.1%, *p* = 0.0024).

These remarkable improvements in survival rates in Florida for the FPBCC centers over the two periods (2016–2018 and 2019–2021) cannot be solely attributed to the establishment of FPBCC and its associated activities. The improvements are also related to general advancement in the field, a better understanding of donor selection, the use of new drugs, improved supportive care, and treatments, and the increased use of post‐transplant cyclophosphamide. Those improvements were also implemented by other centers, reflecting significant improvement in 1‐year survival in other small transplant programs. Additionally, factors that could have decreased survival, such as COVID‐19 impact and staffing shortages, were likely present for all centers, small and large, across the country.

It is believed that the establishment of the FPBCC had an impact on the larger magnitude of improvements. One of the initial focal activities of FPBCC was to better understand outcomes, as demonstrated by prior outcome‐based publications looking at retrospective data (FPBCC prior publications). The ability of the consortium to analyze outcomes data retrospectively for all centers together and identify trends in outcomes helped identify areas for improvement. It also facilitated group discussions on how to implement change.

As previously published, our haploidentical donor transplant recipients were doing better than other mismatched donor transplant recipients [Pediatric HSCT in Florida (2014–2016)], providing support to continue with the selection of haploidentical donors. However, we determined that programs needed to update institutional guidelines for donor selection, and we identified that more detailed reporting of cause of death for patients was needed. These findings and subsequent actions helped programs better select donors and understand better the causes of death. Analyzing the trends in survival before and after consortium activities, it appears that the establishment of the FPBCC has had a positive impact on overall survival, which was the initial main objective of the consortium.

We also noted that the number of transplants performed in FPBCC centers and other small centers decreased over the two time periods (2016–2018 and 2019–2021). For FPBCC centers, the numbers of transplants done over the first 3‐year reporting period was an average of 40.4 ± 8.7, compared to 35 +/−6.5 in the second reporting period, with no statistical difference noted. While the decline seen in small centers (42.6 vs. 38.5) was statistically significant with a *p*‐value of 0.0266, the large centers (124.8 vs. 124.1) did not have a significant decline in transplant numbers. The decline appreciated by FPBCC centers can be attributed to similar reasons that can be attributed to the decline in other small HSCT centers. The reduction of transplant numbers seen in smaller centers could be attributed to the increased use of CAR‐T therapy in patients with relapsed and refractory B‐cell ALL and the potential effect of COVID‐19 in reducing the number of “elective transplants” such as those patients with sickle cell disease or thalassemia.

Overall, small transplant programs, including those in the FPBCC, have made more improvements in 1‐year survival outcomes than the large transplant centers during the same period, 2016–2018 and 2019–2021. This is an impactful and meaningful improvement, which demonstrates that complex transplants can be performed in smaller transplant centers just as safely and effectively as in larger transplant centers, without compromising on outcomes. This will allow families to remain closer to their homes, avoiding additional disruptions in their routines and family structure.

The sharing of knowledge and experience within the FPBCC over this 3‐year intervention period has allowed its members to match the 1‐year survival figures of other leading transplant centers.

## 5 Conclusion

The FPBCC was established in 2018 with the goals of analyzing transplant outcomes and improving survival. Reflecting on survival pre‐ and post‐FPBCC activities have indicated that the activities designed and conducted by the FPBCC were able to significantly improve its 1‐year survivorship outcomes.

The blueprint for the improvement developed by the FPBCC is applicable to other conditions. Our collaborative activities, sharing of knowledge and data, have allowed for significant improvements over a three‐year period among FPBCC centers. This initial approach was geared toward achieving catch‐up improvements. Our next goal will be to develop the disease‐specific expertise of consortium members and to continue to improve survival through clinical trials.

## Data Availability

The data that support the findings of this study are available from the corresponding author upon reasonable request.
